# Comparison of a ceiling-mounted 3D flat panel detector vs. conventional intraoperative 2D fluoroscopy in plate osteosynthesis of distal radius fractures with volar locking plate systems

**DOI:** 10.1186/s12891-021-04784-7

**Published:** 2021-11-02

**Authors:** Raphael Seuthe, Andreas Seekamp, Bodo Kurz, Julian Pfarr, Jost Philipp Schaefer, Simon Peh, Sebastian Lippross

**Affiliations:** 1 Department of Trauma and Orthopedic Surgery, University Medical Center of Schleswig-Holstein, Campus Kiel, Arnold-Heller-Str. 3, 24105 Kiel, Germany; 2 Department of Anatomy, Christian-Albrechts-University, Kiel, Germany; 3 Department of Radiology and Neuroradiology, University Medical Center of Schleswig-Holstein, Campus Kiel, Kiel, Germany

**Keywords:** Distal radius fracture, Insufficient fracture reduction, Plate misplacement, Screw misplacement, Intraarticular screw, Protruding screw, Flat panel detector, Intraoperative 3D fluoroscopy, Hybrid operating room

## Abstract

**Objectives:**

To compare intraoperative 3D fluoroscopy with a ceiling-mounted flat panel detector in plate osteosynthesis of distal radius fractures (AO/OTA 2R3C1.2) with volar locking plate systems to conventional 2D fluoroscopy for detection of insufficient fracture reduction, plate misplacement and protruding screws.

**Methods:**

Using a common volar approach on 12 cadaver forearms, total intraarticular distal radius fractures were induced, manually reduced and internally fixated with a 2.4 distal radius locking compression plate. 2D (anterior-posterior and lateral) and 3D (rotational) fluoroscopic images were taken as well as computed tomographies. Fluoroscopic images, Cone Beam CT (CBCT), 360° rotating sequences (so called “Movies”) and CT scans were co-evaluated by a specialist orthopedic surgeon and a specialist radiologist regarding quality of fracture reduction, position of plate, position of the three distal locking screws and position of the three diaphyseal screws. In reference to gold standard CT, sensitivity and specifity were analyzed.

**Results:**

“Movie” showed highest sensitivity for detection of insufficient fracture reduction (88%). Sensitivity for detection of incorrect position of plate was 100% for CBCT and 90% for “Movie.” For intraarticular position of screws, 2D fluoroscopy and CBCT showed highest sensitivity and specifity (100 and 91%, respectively). Regarding detection of only marginal intraarticular position of screws, sensitivity and specifity of 2D fluoroscopy reached 100% (CBCT: 100 and 83%). “Movie” showed highest sensitivity for detection of overlapping position of screws (100%). When it comes to specifity, CBCT achieved 100%. Regarding detection of only marginal overlapping position of screws, 2D fluoroscopy and “Movie” showed highest sensitivity (100%). CBCT achieved highest specifity (100%).

**Conclusion:**

As for assessment of quality of fracture reduction and detection of incorrect position of plate as well as overlapping position of the three diaphyseal screws CBCT and “Movie” are comparable to CT – especially when combined. Particularly sensitivity is high compared to standard 2D fluoroscopy.

## Introduction

Distal radius fractures are amongst the most common fractures [1, 2]. In recent studies, they accounted for estimated 19% of all incident fractures and occasioned 3% of the total costs of over 16.9 billion dollars in the United States (US) alone [3] – not taken into consideration decreased school attendance, lost work hours, loss of independence and lasting disability [4]. In the majority of cases, these fractures overtake the elderly and result from low-energy trauma such as falls from a standing height [5]. Bearing in mind the on and on increasing life expectancy, a rising incidence of distal radius fractures can be expected [6].

The volar locking plate system was established in 2000 and has rapidly become gold standard in treatment of distal radius fractures [7–10]. It is a safe procedure, offering biomechanically stable fixation and allowing early rehabilitation [6, 11]. Major complications – amongst others – are early posttraumatic arthrosis in the radiocarpal joint and irritations/ruptures of flexor and extensor tendons. The former derives from insufficient fracture reduction [12, 13] and intraarticular positioned screws [8, 14], the latter from plate misplacement (especially ruptures of the flexor pollicis longus tendon) [15, 16] or dorsally protruding screws (especially ruptures of the extensor pollicis longus tendon) [8, 14, 17]. To correct these mistakes intraoperatively and avoid postoperative CT and revision surgery, the surgeon needs competent knowledge of the complex distal radius anatomy [7, 18] and high-quality imaging [1, 8].

Conventional intraoperative 2D fluoroscopy – even when performing additional views – does not detect screw misplacements reliably [19–21]. Thus, additional imaging modalities were used lately [14, 22]. Intraoperative computed tomography is a conceivable option [7, 23], but not yet widely available. Intraoperative 3D fluoroscopy however can be performed with almost every up-to-date mobile image intensifier and has proven its benefits in various anatomical regions allowing the surgeon prompt correction of insufficient fracture reductions or plate/screw misplacements [18, 24–28].

In recent years, hybrid operating rooms have been installed widely. Originally designed for cardiac and vascular interventions, they nowadays are more and more used interdisciplinarily [13, 24, 29–35]. In 2014, the operation center (OPZ) of the University Medical Center Schleswig-Holstein, Campus Kiel was equipped with a ceiling-mounted flat panel detector (Allura Xper FD 20 with FlexMove, Philips, Best, The Netherlands). The system has – compared to other systems [36, 37] – a relatively large detector (30x40cm) and is able to take a series of 230 2D images by performing a rotational scan. From these images, a 360° rotating sequence (so called “Movie”) and a CT-like 3D volumetric view (so called Cone beam CT (CBCT)) can be generated. Both modalities can be immediately displayed to the surgeon on a 58″ monitor also mounted to the ceiling.

The purpose of this study is to show that, by using Allura Xper FD 20 with FlexMove in plate osteosynthesis of distal radius fractures with volar locking plate systems, insufficient fracture reduction, plate misplacement and protruding screws are more likely to be discovered intraoperatively and therefore corrected immediately than by using conventional intraoperative 2D fluoroscopy alone. The need for postoperative CT and revision surgery can be reduced.

## Materials and methods

All methods were carried out in accordance with relevant guidelines and regulations. Ethics approval was obtained from the Ethics committee of the Christian-Albrecht-University Kiel, Schleswig-Holstein, Germany, Number AZ: D 485/18. All donors underwent informed consent for the use of their cadavers in the anatomical department.

### Surgical preparation

The surgical preparations took place at Christian-Albrechts-University, Department of Anatomy, Kiel. A total of 12 cadaver forearms were prepared. Using numbers provided by the Department of Anatomy preserved the donators’ anonymity and guaranteed the discriminability of the specimens.

After having performed a common volar approach, a 22 mm Stille type chisel and a 3 kg hammer were used to conduct the osteotomy. In order to mimic a total intraarticular distal radius fracture (AO/OTA 2R3C1.2), a coronal and an intraarticular sagittal osteotomy were induced.

Following manual reduction, a 2.4 distal radius locking compression plate (LCP, DePuy Synthes Companies of Johnson & Johnson, Norderstedt, Germany) was used for internal fixation. Three locking screws were placed in the distal fragments and one cortical and two locking screws were inserted into the diaphysis, always using a depth gauge to measure for screw length.

### Imaging procedures

**Fig. 1:**
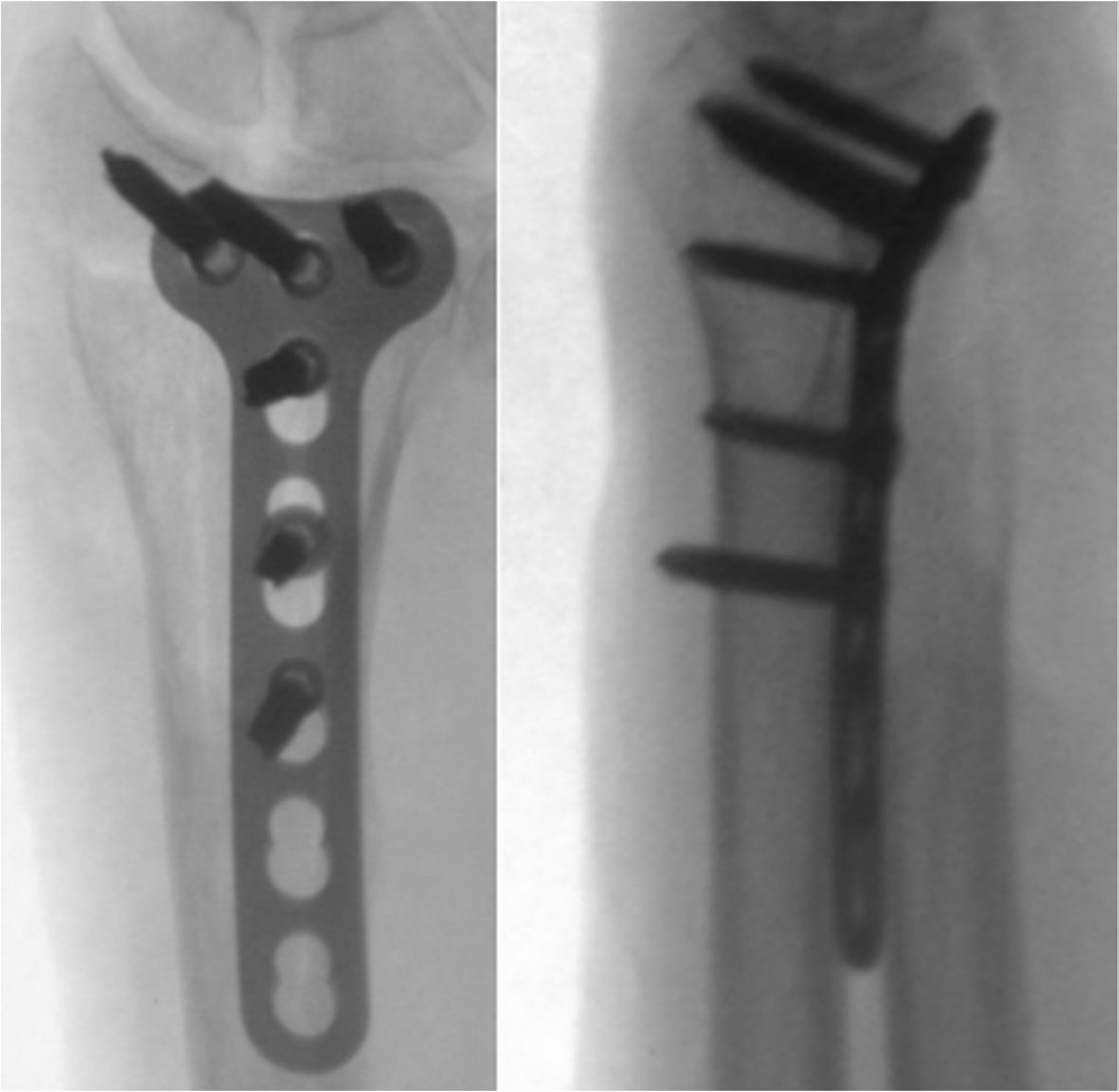
2D Fluoroscopy in anterior-posterior and lateral planes

Fluoroscopic images in anterior-posterior and lateral planes were taken at Department of Anatomy using an Expo 8000 image intensifier (Ziehm, Erlangen, Germany; see Fig. 1).

**Fig. 2:**
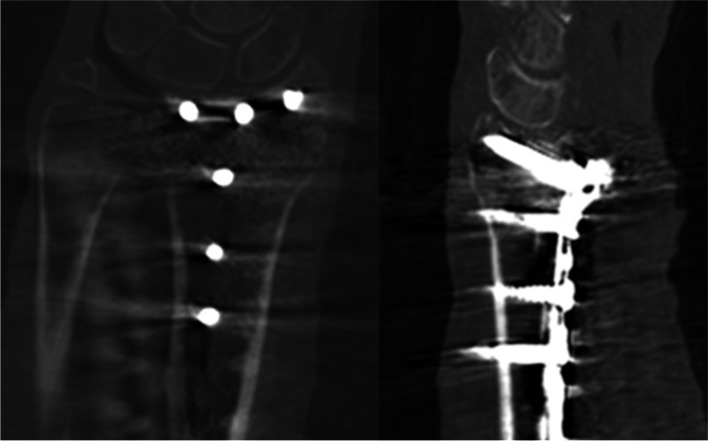
CBCT images in coronal and sagittal planes

**Fig. 3:**
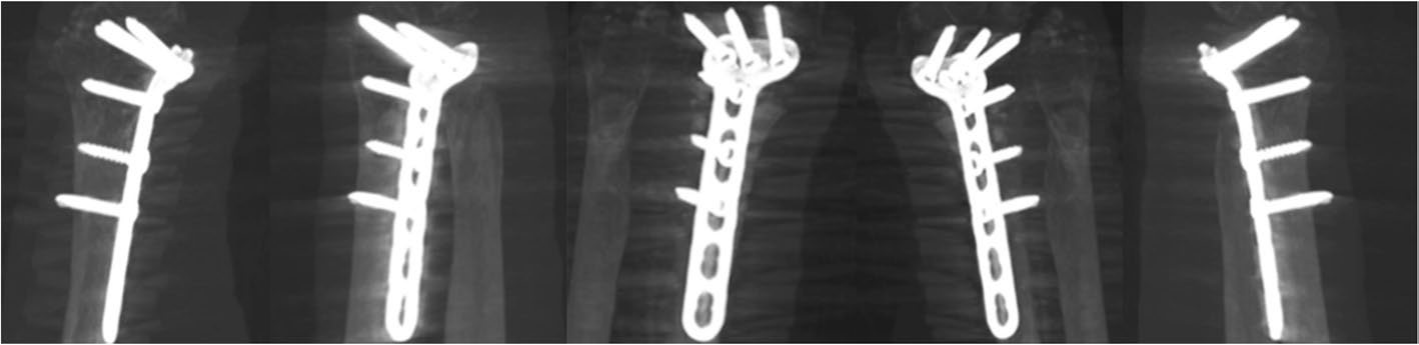
“Movie” sequence: a sample of 5 images (angle range 190°, 230 single images)

**Fig. 4:**
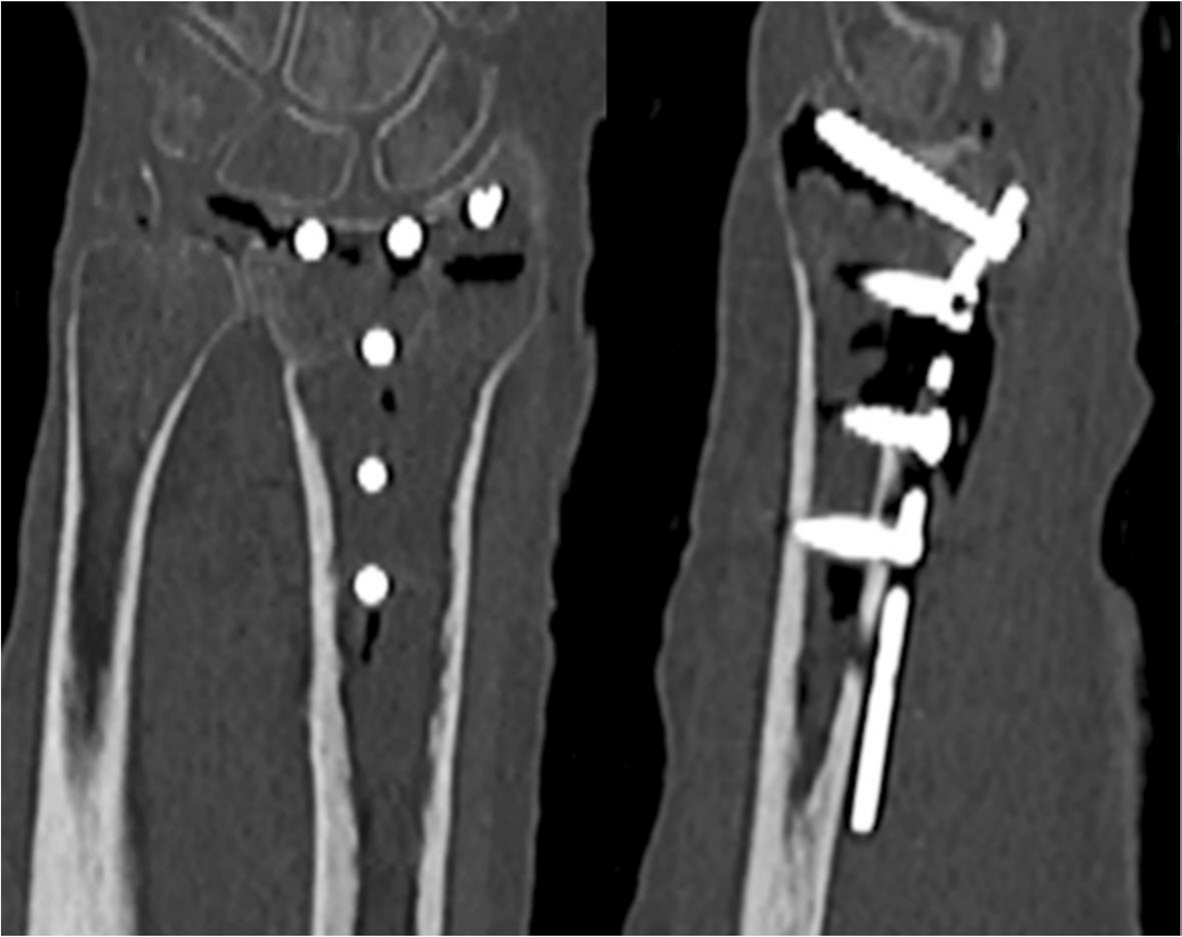
CT images in coronal and sagittal planes

Rotational scans and computed tomographies were taken at the University Medical Center of Schleswig-Holstein, Campus Kiel – the rotational scans in the hybrid operating room, the computed tomographies at Department of Radiology. For rotational scans we used the above-mentioned Allura Xper FD 20 with FlexMove and its protocol for small bones. The respective specimen was placed on the operating table in the system’s isocenter in anterior-posterior direction. After a positive test run with anti-collision device activated, the latter was deactivated and the scan performed (rotation speed 30°/sec, angle range 190°, 230 single images, rotation time with acceleration and deceleration approximately 8 s). CBCT 3D-reconstruction (see Fig. 2) and the “Movie” called sequence (see Fig. 3) were displayed on the 58″ ceiling-mounted monitor within seconds. CT scans were performed using a Somatom Definition Flash with Dual Energy acquisition technique (Siemens Healthcare GmbH, Erlangen, Germany) and its protocol for the distal forearm (see Fig. 4).

### Data evaluations

2D Fluoroscopic images, CBCT reconstructions, “Movie” sequences and CT scans were co-evaluated by a specialist orthopedic surgeon (more than 10 years of professional experience) and a specialist radiologist (6 years of professional experience) neither of which was involved in surgical preparations or imaging procedures. Consensus had to be achieved. In four weekly sessions 12 of the overall 48 image series were evaluated at a time in random order. To avoid recognitions, only one modality per specimen per session was assessed. Parameters to be evaluated were:

- quality of fracture reduction (1: sufficient; 2: fracture gap too wide),
- position of plate (1: correct; 2: acceptable: the position of plate does not comply with the manufacturer’s directions, but will provide a stable osteosynthesis anyhow; 3: inacceptable),
- position of the three distal locking screws (0: extraarticular; 1 marginally intraarticular (less than two millimeters over cortical bone surface); 2: intraarticular),
- position of the three diaphyseal screws (overlapping of 2nd cortical bone; 0: not overlapping; 1: marginally overlapping (less than two millimeters over cortical bone surface); 2: overlapping).

### Descriptive statistics

Due to the relatively small number of specimens, descriptive statistics was the method of choice. If there were three assessment criteria (e.g. in plate positioning), the resulting 3 × 3 cross tabulation had to be divided into two 2 × 2 cross tabulations to allow a statistical evaluation for sensitivity and specifity. In reference to gold standard CT, true positive (TP), false negative (FN), true negative (TN) and false positive (FP) cases were listed for 2D fluoroscopy, CBCT and “Movie”. Finally, sensitivity (TP/(TP + FN)) and specifity (TN/(TN + FP)) were analyzed.

## Results

### Quality of fracture reduction

“Movie” showed highest sensitivity for detection of insufficient fracture reduction (88%). All three methods achieved 100% for specifity (see Table 1 for details).

**Table 1:** Sensitivity and Specifity for quality of fracture reduction in relation to CT (gold standard)

	True positive	False negative	True negative	False positive	Sensitivity (%)	Specifity (%)
2D Fluoroscopy	6	2	4	0	**75**	**100**
“Movie”	7	1	4	0	**88**	**100**
CBCT	5	3	4	0	**63**	**100**

### Position of plate

Sensitivity for detection of incorrect position of plate was 100% for CBCT and 90% for “Movie” (see Table 2). Regarding specifity for detection of correct position of plate and sensitivity and specifity for detection of acceptable position of plate, there were no differences between the three methods (100%, see Table 3).

**Table 2:** Sensitivity and Specifity for position of plate in relation to CT (gold standard)

	True positive	False negative	True negative	False positive	Sensitivity (%)	Specifity (%)
2D Fluoroscopy	5	3	2	0	**63**	**100**
“Movie”	9	1	2	0	**90**	**100**
CBCT	10	0	2	0	**100**	**100**

**Table 3:** Sensitivity and Specifity for acceptable position of plate in relation to CT (gold standard)

	True positive	False negative	True negative	False positive	Sensitivity (%)	Specifity (%)
2D Fluoroscopy	2	0	1	0	**100**	**100**
“Movie”	1	0	1	0	**100**	**100**
CBCT	2	0	1	0	**100**	**100**

### Position of the three distal locking screws

For intraarticular position of screws, 2D fluoroscopy and CBCT showed highest sensitivity and specifity (100 and 91%, respectively). Regarding detection of only marginal intraarticular position of screws, sensitivity and specifity of 2D fluoroscopy reached 100% (CBCT: 100 and 83%; see Tables 4 and 5 for details).

**Table 4:** Sensitivity and Specifity for position of the three distal locking screws in relation to CT (gold standard)

	True positive	False negative	True negative	False positive	Sensitivity (%)	Specifity (%)
2D Fluoroscopy	27	0	10	1	**100**	**91**
“Movie”	27	2	6	3	**93**	**67**
CBCT	27	0	10	1	**100**	**91**

**Table 5:** Sensitivity and Specifity for marginal intraarticular position of the three distal locking screws in relation to CT (gold standard)

	True positive	False negative	True negative	False positive	Sensitivity (%)	Specifity (%)
2D Fluoroscopy	1	0	5	0	**100**	**100**
“Movie”	0	1	3	3	**0**	**50**
CBCT	1	0	5	1	**100**	**83**

### Position of the three diaphyseal screws

“Movie” showed highest sensitivity for detection of overlapping position of screws (100%; CBCT: 95%). When it comes to specifity, CBCT achieved 100%, “Movie” 94% and 2D fluoroscopy 83%. Regarding detection of only marginal overlapping position of screws, 2D fluoroscopy and “Movie” showed highest sensitivity (100%). CBCT achieved highest specifity (100%; see Tables 6 and 7 for details).

**Table 6:** Sensitivity and Specifity for position of the three diaphyseal screws (overlapping of 2nd cortical bone) in relation to CT (gold standard)

	True positive	False negative	True negative	False positive	Sensitivity (%)	Specifity (%)
2D Fluoroscopy	16	4	24	5	**80**	**83**
“Movie”	19	0	30	2	**100**	**94**
CBCT	18	1	34	0	**95**	**100**

**Table 7:** Sensitivity and Specifity for position of the three diaphyseal screws (marginal overlapping of 2nd cortical bone) in relation to CT (gold standard)

	True positive	False negative	True negative	False positive	Sensitivity (%)	Specifity (%)
2D Fluoroscopy	4	0	12	4	**100**	**75**
“Movie”	5	0	15	1	**100**	**94**
CBCT	4	1	17	0	**80**	**100**

## Discussion

Here we describe the use of a modern imaging suite in a hybrid operating room that has potential to bring great value for the use in orthopedics and hand surgery. The Allura Xper FD 20 with FlexMove with its mobile table and ceiling-mounted large C-arm can not only record and store 2D fluoroscopic images, but also immediately provide CBCT 3D-reconstruction and the “Movie” called sequence to the surgeon.

The standard intraoperative procedure applied by most surgeons to verify fracture reduction as well as screw and plate positioning is fluoroscopy in two planes. Only in case of clinical signs of insufficient fracture reduction or screw or plate misplacement a postoperative CT scan is requested. This bears the risk of having to perform revision surgery [38]. Some improvement in detection of the above mentioned complications can be achieved by taking additional fluoroscopy planes, including the so-called “skyline view”, “radial groove view” and “carpal shoot through view.” [8, 19, 39–44] However, to this day a reliable assessment with fluoroscopy alone is not possible [21]. Others try to implement ultrasound [45–47] or suggest the arthroscopic controlled fracture reduction [48–50].

We examined – according to our hypothesis – if, by using Allura Xper FD 20 with FlexMove, insufficient fracture reduction, plate misplacement and protruding screws are more likely to be discovered intraoperatively when perfoming plate osteosynthesis of distal radius fractures with volar locking plate systems than by using conventional intraoperative 2D fluoroscopy only. Therefore, we compared the subjective consensus-ratings of two specialists (regarding quality of fracture reduction, position of plate, position of the three distal locking screws and position of the three diaphyseal screws) for the three modalities 2D fluoroscopy, “Movie” and CBCT against the gold standard CT.

As far as quality of fracture reduction is concerned, “Movie” (88%) is more sensitive than 2D fluoroscopy (75%). A possible explanation could be that the sequence provides a large series of multiple planes (230 over 190°) which results in an easier detection of gaps and steps. In our experience, the “Movie” creates a deeper understanding of the fracture’s morphology in the surgeon’s imagination.

As for detection of correct position of plate, CBCT is as sensitive as CT. With its sensitivity of 90%, “Movie” is not far apart from gold standard as well. We assume the 3D capabilities of all three modalities helped in the assessment.

Surprisingly, there was no difference in sensitivity and specifity for detection of intraarticular position of the three distal locking screws between CBCT and 2D fluoroscopy (both 100 and 91%, respectively). A possible explanation could be the fact that the two co-evaluating specialists are well trained in detecting intraarticular screws in fluoroscopic images. Furthermore, CBCT is known to be prone to metal artifacts [36]. Like others, we experienced quite a few metal artifacts in the CBCT reconstructions.

Finally, CBCT (95%; 100%) and “Movie” (100%; 94%) were far more sensitive and specific in detection of position of the three diaphyseal screws than 2D fluoroscopy (80%; 83%). Again, the 3D capabilities of CBCT and “Movie” allowed the two specialists to exactly visualize the complex and individual anatomy (in particular the dorsal tubercle of radius/Lister’s tubercle) of the distal radius. Maybe the fluoroscopy’s results could have been improved by taking additional planes.

In both, position of the three distal locking screws and position of the three diaphyseal screws, we drew a line between “marginally intraarticular/intraarticular” and marginally overlapping/overlapping” at 2 mm. This derived from our own experience and may be subject for further debate.

There are a few limitations: First, there is our setup with anatomic cadaver specimens rather than a real surgical setting. Therefore, no clinical information was obtainable. Nevertheless, this setup ensured a standard procedure especially in terms of type of fracture and surgical approach. We consent with Beisemann et al. that improved reposition and enhanced implant positioning will influence the clinical outcome favorably [24]. Besides, we cannot make a point regarding the average operating time in a clinical setting. Others report extended operating times between 5 and 10 min when using 3D fluoroscopy [8, 18, 51, 52].

Another downside of our study was the small number of specimens. This limits the statistic power of the data and allowed descriptive statistics only. Unfortunately, there were no more specimens available at the time.

The data was evaluated by a specialist orthopedic surgeon and a specialist radiologist. They both are well trained in evaluating fluoroscopic images and CT reconstructions, but to a lesser extent in the evaluation of CBCT reconstructions and “Movie” sequences. Furthermore, a specialist radiologist is usually not a member of the surgical team. Thus, the evaluation didn’t reflect the common intraoperative situation.

All imaging modalities underly some subjective bias and error. Even the gold standard CT may be misinterpreted and there will always remain some uncertainty regarding the accuracy of ratings.

Finally, we did not examine the potential intraoperative radiation exposure of the patient or the surgical team (including the anesthetist) [53] nor did we include the total costs of the method. In terms of radiation exposure of the patient one has to bear in mind that a postoperative CT can be avoided nine times out of ten by using our method. In terms of radiation exposure of the surgical team: There is no additional radiation exposure because the team members usually leave the operating room during the 8 s CBCT scan [18]. We agree with Richter and Gebhard that the interdisciplinary use of hybrid operating rooms makes them time- and cost-effective [54] – at least compared to postoperative CT [55].

## Conclusion

As for assessment of quality of fracture reduction and detection of correct position of plate as well as position of the three diaphyseal screws CBCT and “Movie” are comparable to CT – especially when combined. Particularly sensitivity is high compared to standard 2D fluoroscopy.

## Data Availability

The datasets used and/or analysed during the current study available from the corresponding author on reasonable request.
